# Notes on a Case of Syphilitic Cleft Palate, with Its Treatment

**Published:** 1890-07

**Authors:** E. Lloyd-Williams

**Affiliations:** Assistant Dental Surgeon to the Dental Hospital of London.


ARTICLE V.

### NOTES ON A CASE OF SYPHILITIC CLEFT PALATE, WITH ITS TREATMENT.

BY E. LLOYD-WILLIAMS, L.R.C.P., LOND., M.R.C.S., L.D.S., ENG.
[Assistant Dental Surgeon to the Dental Hospital of London.]

A woman, 37 years of age, applied at the Dental Hospital of London for relief under the following circumstances. About seventeen years ago she had acquired syphilis, which in its tertiary stage had caused severe ulceration in the mouth and throat. Upon examination, all active ulceration appeared to have ceased, the soft tissues being firm and healthy. The hard palate was extremely cleft from a point immediately behind the anterior palatine canal, a large quantity of bone having been removed as a result of the syphilitic periostitis; there was also a large gap in the alveolar border anterior to the cleft. The mucous membrane covering the hard palate was intact with the exception of an opening the size of a goose-quill at about the middle of the palate, and one somewhat larger, anterior to it. The soft palate was very slightly split from below, through, and as far as the base of the uvula, while the free edges were glued to the walls of the pharynx. The nose was thus completely cut off from the pharynx posteriorly, with the exception of a small opening where the soft palate was fissured. One sound bicuspid remained, all the rest of the teeth being either absent, or represented by loose and unhealthy stumps. The loose stumps having been removed, and the alveolar tissues brought into a healthy condition, the question remained as to what might be done to relieve the patient in speaking, eating, and drinking. It was doubtless desirable, in order to improve the vocal articulation, as well as to make swallowing more easy, that the soft palate should, if possible, be freed from its attachment to the pharynx; and on this point I obtained the opinion of Mr. Bland Sutton, who thought that there was very little chance of successful interference.

It was then decided to afford what relief was possible by mechanical means. An impression was attempted in plaster of Paris, with most disastrous results; and as the accident was a somewhat uncommon one, an account of it may be of some slight value as a warning to others. The tray was an extemporized one, formed by cutting out a wax impression in an ordinary tray; into this the plaster was poured and pressed into place. When the impression was removed, it was found that the plaster had run through the two openings in the palate, and becoming confluent, formed a fairly large mass which was retained within the nose. The mass was split up as well as possible into small fragments, and by means of forceps, syringe, &c., several pieces were removed. The patient departed, and when next seen, complained of considerable inconvenience from a foreign body in the nose. On examination it was found that a tolerably large lump of plaster still remained imprisoned. As it was difficult to seize and crush, it was decided to divide the soft tissues between the two openings, thus forming one larger orifice. This was done, but the bony edges of the cleft prevented the escape of the plaster. Crushing was then resorted to, followed by syringing strong streams of water in all directions. Tampons of cotton-wool were eventually passed through the narrow pharyngeal opening, in both directions, but all to no avail with regard to one piece which evaded all our ingenuity, so the patient, being thoroughly exhausted, was once more dismissed. Four days later a happy solution of the difficulty occurred by the patient finding the piece of plaster in her mouth. I need scarcely add that nothing will induce me in the future to use plaster for impression of this description.

Ultimately an impression was taken in composition, and a very light vulcanite denture made, which not only acted as an obturator, but also proved of great comfort both in speaking and eating. At the part where the loss of alveolus was most pronounced, the plate was made hollow; and as this method of lightening vulcanite dentures is useful in a large number of ordinary cases, the *modus operandi* may be described for the benefit of those who may not be acquainted with it.

The plaster model is carefully dried, but the oral surface, untouched by stearine or any other hardening agent, must be kept clean and free from dirt or grease. The case is now built up in the ordinary way in wax, and flasked in *three parts*—the only safe method with which I am acquainted—the original model in the lower part, the teeth and labial aspect of gum, embraced by the middle part, and the palate covered in by the third part of the flask or *plug*. In other words, this method of flasking is the *contour* method *plus* the splitting up of the first part of the investment into two operations. Almost any ordinary roomy flask may be made available, if the lid be drilled and a good strong tag riveted on to it. Having boiled out the wax and dried thoroughly, the pink gum is carefully packed all round the teeth in the *second* part of the flask, and put away to keep just warm. The first part is now taken and the model painted with a solution of the rubber to be used in methylated chloroform. At the spot where the hollow chamber is desired a thin layer of rubber is pressed over the surface with the fingers, and then with hot smooth oval-surfaced packer shaped into a deep saucer-like cavity, the surface being made sure by a layer of rubber solution. The first and second parts of the flask are now brought together in a vice and secured by pins or wedges. The remainder of the packing is now done from the palate, the contouring being complete except the lid of the hollow chamber. Having cut an overplus channel at the heel of the plug, paint the surface of the plug *opposite* the lid of the chamber with rubber solution, and press on one thickness of rubber. Place two or three drops of water in the cavity; put in the plug, avoiding pressure; keep in an upright position whilst placing in vulcanizer; and screw home when the gauge stands at 25 lbs. If these steps are carefully taken various chambers of different shapes may be made in ordinary edentulous cases, when the matter of weight—and sometimes porosity of rubber—become a serious consideration. The method of flasking and packing described is in my opinion the only one for general purposes which is absolutely reliable. Properly carried out it become impossible to raise the bite, or to produce that *piebald* appearance of gum caused by the erratic wanderings of rubber, which is *not* artistic although common, and which patients unfortunately sometimes take exception to. I know it is dangerous—especially in mechanical matters—to appear to dogmatize on a subject to experienced persons, but perhaps some of the younger students who read this may care to try a method of flasking which will save them a good deal of heart-burning in the future days of busy practice.—*London Dental Record.*

